# Lymphotoxin-α Gene and Risk of Myocardial Infarction in 6,928 Cases and 2,712 Controls in the ISIS Case-Control Study

**DOI:** 10.1371/journal.pgen.0020107

**Published:** 2006-07-07

**Authors:** Robert Clarke, Peng Xu, Derrick Bennett, Sarah Lewington, Krina Zondervan, Sarah Parish, Alison Palmer, Sarah Clark, Lon Cardon, Richard Peto, Mark Lathrop, Rory Collins

**Affiliations:** 1 Clinical Trial Service Unit, Old Road Campus, Oxford, United Kingdom; 2 Centre National de Genotypage, Paris, France; 3 Wellcome Trust Centre for Human Genetics, Old Road Campus, Oxford, United Kingdom; North Carolina State University, United States of America

## Abstract

Lymphotoxin-α (LTA) is a pro-inflammatory cytokine that plays an important role in the immune system and local inflammatory response. LTA is expressed in atherosclerotic plaques and has been implicated in the pathogenesis of atherosclerosis and coronary heart disease (CHD). Polymorphisms in the gene encoding lymphotoxin-α *(LTA)* on Chromosome 6p21 have been associated with susceptibility to CHD, but results in different studies appear to be conflicting. We examined the association of seven single nucleotide polymorphisms (SNPs) across the *LTA* gene, and their related haplotypes, with risk of myocardial infarction (MI) in the International Study of Infarct Survival (ISIS) case-control study involving 6,928 non-fatal MI cases and 2,712 unrelated controls. The seven SNPs (including the rs909253 and rs1041981 SNPs previously implicated in the risk of CHD) were in strong linkage disequilibrium with each other and contributed to six common haplotypes. Some of the haplotypes for *LTA* were associated with higher plasma concentrations of C-reactive protein (*p* = 0.004) and lower concentrations of albumin (*p* = 0.023). However, none of the SNPs or related haplotypes were significantly associated with risk of MI. The results of the ISIS study were considered in the context of six previously published studies that had assessed this association, and this meta-analysis found no significant association with CHD risk using a recessive model and only a modest association using a dominant model (with narrow confidence intervals around these risk estimates). Overall, these studies provide reliable evidence that these common polymorphisms for the *LTA* gene are not strongly associated with susceptibility to coronary disease.

## Synopsis

Lymphotoxin-α gene *(LTA)* encodes a cytokine involved in the immune system that has been linked with risk of coronary heart disease (CHD). In one of the first genome-wide association studies, a Japanese group reported that polymorphisms for the *LTA* gene were associated with a 1.8-fold higher risk of CHD. The authors of this current paper examined associations of several polymorphisms for the *LTA* gene with risk of CHD in a case-control study of 6,928 heart attack survivors and 2,712 unrelated controls in the United Kingdom. None of the polymorphisms for *LTA* (including those examined in the original Japanese study) were associated with CHD risk. Moreover, a meta-analysis of all published studies found no significant association of *LTA* with CHD risk. These findings emphasize the need for large-scale studies to have sufficient power to detect associations of genetic variants with CHD risk and the need for replication of any such associations in independent studies of even larger size.

## Introduction

Lymphotoxin-α (LTA) is a cytokine produced by all cells in the body in response to tissue injury, and it plays an important role in regulation of the immune system and local inflammatory response [1]. *LTA* and tumour necrosis factor share a common receptor that controls the expression of vascular endothelial cell adhesion molecules and nitric oxide production [2]. LTA is expressed in atherosclerotic plaques in animal models, and has been implicated in the pathogenesis of atherosclerosis and coronary heart disease (CHD) [3,4].

In an initial genome-wide case-control screen involving 65,671 single nucleotide polymorphisms (SNPs) from 13,738 genes in 94 myocardial infarction (MI) cases and 658 controls in Japan, susceptibility to MI appeared to be associated with the SNP 252A→G (rs909253) in the *LTA* gene, which encodes LTA on Chromosome 6p21 [5]. This possible association with rs909253, and with two further SNPs (rs1800683 and rs1041981) in near-perfect linkage disequilibrium (LD) with it, was subsequently replicated by the same investigators in a further 1,133 MI cases and 1,006 controls. Among these SNPs, the non-synonymous coding SNP rs1041981 seemed to be most strongly implicated because of its association with induction of vascular endothelial adhesion molecules [5]. An association of rs909253 or rs1041981 with the risk of CHD has subsequently been observed in some smaller studies [6,7], but not in others [8–10].

If genetic variants associated with the control of pro-inflammatory cytokines are associated even modestly with MI risk, this could have important implications for understanding the pathogenesis of atherosclerosis. However, reliable confirmation or refutation of such associations typically requires studies involving much larger numbers of cases than in the original hypothesis-generating studies [11,12]. We have examined the association between seven SNPs in the *LTA* gene, and their related haplotypes, with risk of MI in the large ISIS [13] case-control study, and then included it in a meta-analysis of all previously published reports on this association.

## Results

Table 1 shows the location and relevant characteristics of the seven SNPs in the *LTA* gene on chromosome 6p21 that were examined in the International Study of Infarct Survival (ISIS) study. Data for six of these SNPs were available in 6,928 cases and 2,712 controls, and for one SNP (rs1041981) in only 6,858 cases and 2,690 controls who had sufficient DNA. The mean age was 54.8 y (standard deviation ±7.3 y) in cases and 46.2 y (standard deviation ±9.6 y) in controls (Table 2). The MI cases had a higher mean body mass index (BMI) and higher prevalence of current smokers, hypertension, and diabetes mellitus compared with controls. Table 2 also shows that, compared with controls, MI cases had higher plasma concentrations of apolipoprotein B, fibrinogen, and C-reactive protein (CRP), and lower mean concentrations of apolipoprotein A_1_ and albumin.

**Table 1:**
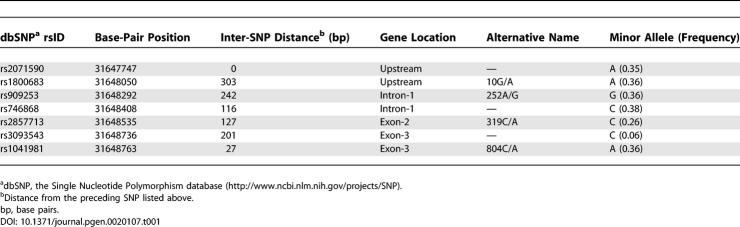
Identity and Base-Pair Position of SNPs for the *LTA* Gene on Chromosome 6, and Minor Allele Frequencies in Controls in the ISIS Study

**Table 2:**
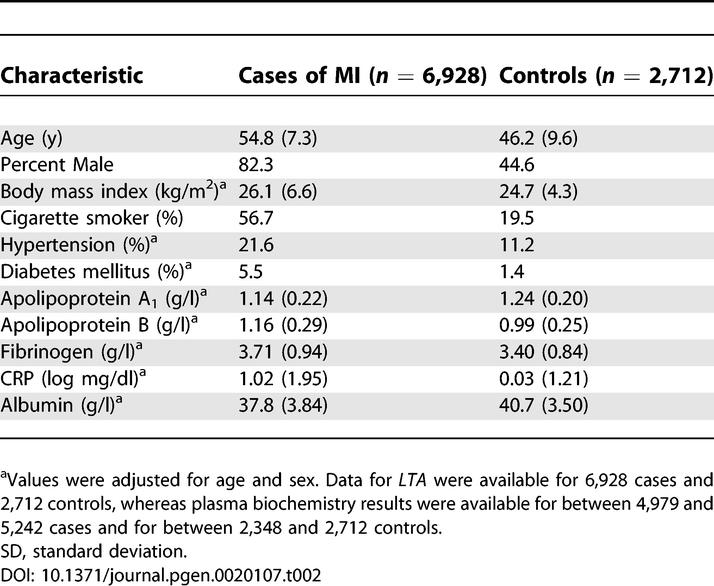
Characteristics of Confirmed Myocardial Infarction Cases and Controls (Mean [SD] or %)

### *LTA* SNPs and Haplotypes

All seven SNPs studied in the *LTA* gene were in Hardy-Weinberg Equilibrium (HWE) among both cases and controls (unpublished data). Reproducibility of the genotyping was high, with a weighted kappa score of 0.99–1.00 for each of these seven SNPs in the *LTA* gene (unpublished data). The region of *LTA* investigated in our study, which spanned about 1 kb and was bounded by rs2071590 and rs1041981, and included all three SNPs examined in the study that had generated the hypothesis of association with CHD [5]. It also included the rs1041981 SNP examined in the PROCARDIS and other published studies of the *LTA* gene and CHD [9,10]. Table 3 shows that all seven SNPs examined were in high LD, with pair-wise LD measured in terms of D^I^ ≥ 0.85 between each SNP. Although the pairwise *r^2^* values between some of the SNPs were low (perhaps due to differences in allele frequency), the *r^2^* values between the rs909253, rs1800683, and rs1041981 SNPs implicated in the hypothesis-generating study [5] were ≥0.98. Hence, all of the SNPs studied were incorporated in the haplotype analysis.

**Table 3:**
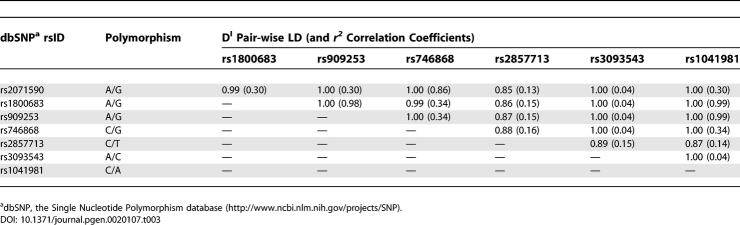
Pair-Wise Standardised Disequilibrium (D^I^) and Correlation *(r^2^)* Coefficients, between Individual SNPs for the *LTA* Gene

### Associations with CRP, Fibrinogen, and Albumin

Table 4 shows the mean (standard error) values of cardiovascular risk factors by individual *LTA* haplotypes in the controls. Using the haplotype-based General Linear Model (GLM), statistically significant differences were observed between these haplotypes and plasma concentrations of CRP and albumin (although the absolute differences were generally small), which is consistent with some of these SNPs being functional. There were no significant differences between the haplotypes in BMI or plasma concentrations of apolipoprotein A_1_, apolipoprotein B, or fibrinogen.

**Table 4:**
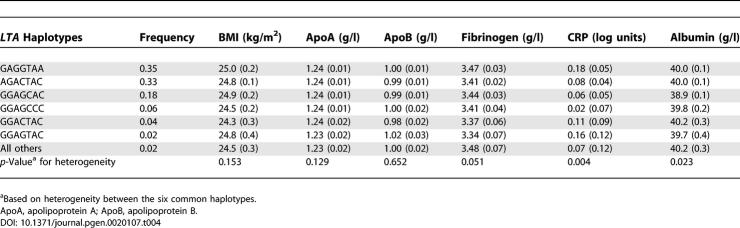
Mean (SE) Values for Selected Characteristics by Haplotypes for the *LTA* Gene in Controls

### Association with MI Risk

Table 5 displays the associations with MI risk in the ISIS case-control study for individual SNPs of the *LTA* gene, after adjustment for age and sex, when analyzed using co-dominant, recessive, and dominant genetic models, respectively. Neither the rs909253 nor the rs1041981 SNP for the *LTA* gene that was studied by Ozaki et al. [5] was significantly associated with MI risk using any of the genetic models. Although the most frequent genotype has been used as the reference category, any group can be compared with any other since each is displayed with its appropriate confidence interval (CI) using “floated” absolute risks (see Materials and Methods). None of the individual haplotypes for the *LTA* gene was associated with MI risk (χ^2^ test of heterogeneity: χ^2^_6_ = 7.97, *p* = 0.24) (unpublished data).

**Table 5:**
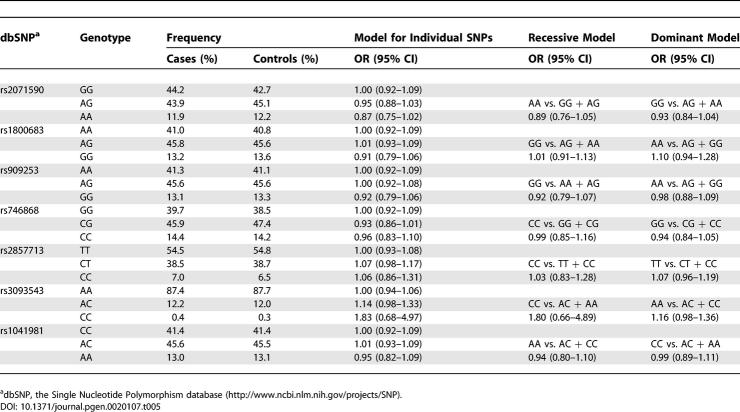
Odds Ratio (95% CI) of MI Associated with SNPs for *LTA* in ISIS Study (Adjusted for Age and Sex)

### Comparison with Other Studies

**Figure 1:**
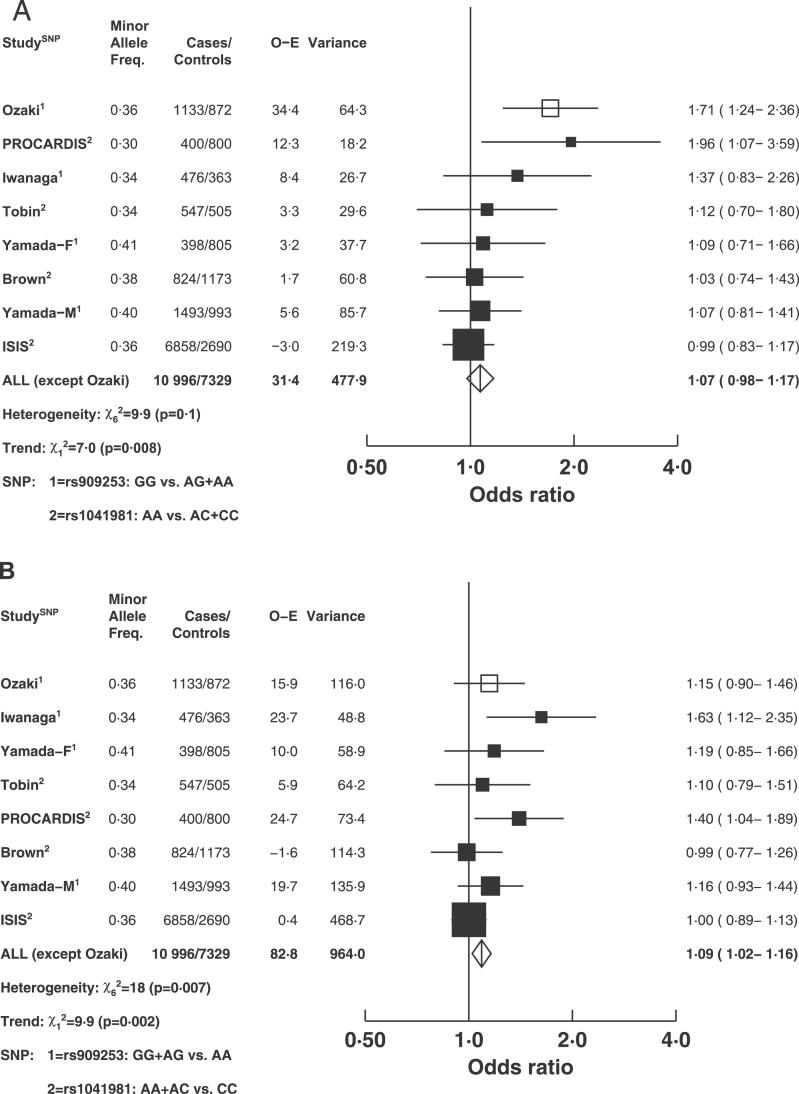
Odds ratio (CI) for Coronary Heart Disease Associated with Genotypes of the *LTA* Gene in ISIS and Other Studies Using Recessive and Dominant Models (A) shows the recessive model and (B) shows the dominant model. Studies are of either the rs909253 SNP (A/G alleles) or the rs1041981 SNP (A/C alleles). The hypothesis-generating study [5] has been excluded from the combined estimates for all other studies that tested the hypothesised association. Size of the squares is proportional to the variance of the log odds ratio, and horizontal lines represent the 99% CIs, and the width of the diamonds represents the 95% CIs. The associations have not been adjusted for age or sex.

Figure 1 shows the associations of CHD risk with rs909253 or rs1041981 in all previously published studies [5–10] and in the present ISIS study. Among the six previous studies, three examined associations with rs909253 [5,6,8] and four examined associations with rs1041981 [5,7,9,10] (and one study reported data separately for males and females [8]). For the ISIS case-control study (without adjustment for age and sex), the association with rs1041981 is displayed in Figure 1, but that SNP was in near perfect LD with rs909253 (which had an odds ratio for MI of 0.99 [99% CI: 0.83–1.17] in a recessive model and 1.00 [99% CI: 0.89–1.13] in a dominant model: see Figure 1). After excluding the hypothesis-generating study [5], the combined odds ratio in a recessive model was 1.07 with a 95% CI of 0.98–1.17 (Figure 1A), and in an autosomal dominant model, it was 1.09 with a 95% CI of 1.02–1.16 (Figure 1B). For the recessive model, there was no significant heterogeneity between the results of the individual studies (χ^2^_6_ = 9.9, *p* = 0.1), although there was a highly significant trend towards the null with increasing study size (χ^2^_1_ = 7.0, *p* = 0.008). For the dominant model, there was significant heterogeneity between the different study results (χ^2^_6_ = 18.0, *p* = 0.007), but this chiefly reflected a trend with study size (χ^2^_1_ = 9.9, *p* = 0.002) and no significant residual heterogeneity (χ^2^_5_ = 8.1, *p* = 0.1). Inclusion of the ISIS results for rs909253 instead of rs1041981 did not change the results of this meta-analysis materially, with a combined odds ratio of 1.06 (95% CI: 0.97–1.16) in the recessive model and 1.08 (95% CI: 1.02–1.15) in the dominant model.

## Discussion

The ISIS study is the largest genetic association study of MI risk and polymorphisms in *LTA,* involving 6,928 MI cases and 2,712 unrelated controls. The recruitment procedure used for controls in the ISIS case-control study (involving spouses of siblings and their children: see Materials and Methods) should have minimised the risk of population admixture between controls and cases. The seven SNPs studied included three that had been previously been reported [5], as well as others identified by resequencing to be in high LD with each other (see Materials and Methods), and covered a genomic region of about 1 kb in the *LTA* gene. The ISIS case-control study found no significant association of MI risk with any of these individual SNPs or with any of the six common haplotypes studied (including those associated with significantly higher plasma concentrations of CRP and albumin).

The present findings differ from those of the hypothesis-generating study [5] which reported that, in a recessive model, the SNPs rs909253 and rs1800683 in the *LTA* gene were associated with odds ratios for MI of 1.71 (95% CI: 1.24–2.36) and 1.78 (95% CI: 1.39–2.27), respectively. That study used a two-stage design in which 65,671 genetic markers in 13,738 genes were initially screened in 94 MI cases and 658 controls, and then the 1% of markers that appeared most strongly associated with MI were re-examined in a further 1,133 MI cases and 1,006 controls. In that study, a third SNP in *LTA* (rs1041981) also appeared to be associated with risk of MI with an odds ratio of 1.78 (95% CI: 1.39–2.27) [5]. It is possible that those results represented false-positive results identified among the relatively small number of cases. Alternatively, it may reflect an imbalance in the control population which was selected from several different population sources, since significant departures from Hardy-Weinberg equilibrium were observed in the genotype distributions in case and control samples in the Ozaki et al. study [5] (possibly indicating population stratification) [14]. Two further case-control studies carried out in Japanese populations have reported conflicting results, with one appearing to confirm an association of the rs909253 SNP with risk of MI [6] and the other not doing so [8].

In a multicentre European study of 400 trio families, the PROCARDIS Consortium reported that rs1041981 was associated in a recessive model with a 1.96 (95% CI: 1.25–3.13) higher risk of CHD before age 65 y [7]. In principle, a study design involving the use of trios should eliminate the potential risk of population admixture that may occur in unrelated case-control studies. However, the apparent association in PROCARDIS could still reflect the play of chance in a study involving relatively small numbers of diseased cases. A subsequent larger, trio family study of 671 UK families reported no significant association of rs1041981 with risk of MI [10].

An alternative explanation for these apparently discrepant findings might be that none of the SNPs in the *LTA* gene studied by Ozaki et al. is causally associated with MI, but are instead in high LD with some other SNP which is present in higher frequency in Japanese than in populations of European descent [12]. In such circumstances, detection of this association via the common *LTA* SNPs would be facilitated in Japanese populations. There would also be a better chance of observing it in trio families of European descent [7], since a sampling strategy based on at least one family member suffering an MI would inflate the frequency of a causal allele that is otherwise rare in the general population. Lack of SNPs in the *LTA* gene that were genotyped in both the European-descentand Japanese populations in HapMap Phase II [15] did not allow us to investigate the possibility of differential LD patterns between the two populations. However, the absence of an association in a large Japanese population by Yamada et al. [8] and in another large UK trio family study [10] does not support this alternative hypothesis. The ISIS study had more than 90% power to detect a 10% higher risk of MI associated with rs909253 and rs1041981 SNPs for the *LTA* gene (assuming an allele frequency of about 0.35) using co-dominant or recessive genetic models. The selection of controls in the ISIS study from spouses of siblings or spouses of children, together with the very small proportion of participants of non–European descent in this study would indicate that the risk of population stratification is small.

A major problem in genetic association studies has been the high proportion of false-positive results suggested by studies involving relatively small numbers of diseased cases [11,12]. Attenuation in the strength of the observed association in larger studies is consistent with publication bias, whereby more extreme results are more likely to be published earlier [16]. The apparent discrepancy between the results of studies of *LTA* and CHD risk reinforces the need for studies involving larger numbers of disease cases, unrelated controls selected from the same population as the cases, and genotyping of markers that provide good coverage of the gene of interest. The ISIS case-control study of nearly 7,000 cases with MI before age 65 y and of family-based controls, considered in the context of previously reported studies of this association, provides reliable evidence that these common polymorphisms for the *LTA* gene are not strongly associated with susceptibility to coronary disease. The present study cannot exclude the possibility of involvement of other variants near the *LTA* gene (e.g., in more distal regulatory regions), which were not considered.

## Materials and Methods

### Study population.

The design of the ISIS case-control study has been described in detail in previous publications [17,18]. Cases genotyped for this study were men aged 30–54 y and women aged 30–64 y, with non-fatal MI confirmed by cardiac enzyme or electrocardiographic criteria (or both). Blood was collected on admission to hospital with their index MI, and a few months later, information was sought from surviving cases about aspects of their previous lifestyle. A similar questionnaire was sent to their siblings and children older than 30 y, and to the spouses of those relatives, with blood sought from all those who completed a questionnaire. Controls genotyped in the present study were spouses of their siblings or children (i.e., unrelated to the MI cases except by marriage) aged 30–64 y, and with no history of MI, angina, or other definite heart disease. Ethnicity was not recorded, but the family-based recruitment strategy should have yielded similar distributions among cases and controls (and, based on other studies conducted by us in these UK hospitals, more than 95% would be expected to be of European descent [19]). Participants provided consent to participate in the study, which had research ethics committee approval.

### Laboratory methods.

DNA was extracted from frozen buffy coat samples using a Taqman and amplifluor technique, as previously described [17,18]. Seven SNPs for *LTA* (rs2071590, rs1800683, rs909253, rs746868, rs2857713, rs3093543, and rs1041981) were selected, including those typed in previous studies, from among all 22 known SNPs for the *LTA* gene in the National Center for Biotechnology Information (NCBI) genome assembly 35. Genotyping using mass spectrometry was carried out at the Centre Nationale de Genotypage (CNG) in Paris without knowledge of disease status, with samples from cases and controls distributed within each 384-well genotyping plate. Internal controls were used to check the consistency of genotyping between plates, and the reproducibility of genotyping was assessed by replicate measurements in a random sample of 250 controls.

Plasma concentrations of apolipoprotein A_1_ and apolipoprotein B were measured at the Clinical Trial Service Unit (CTSU) using Immuno reagents (Vienna, Austria) on Beckman Synchron CX4 and CX5 auto-analysers (Beckman Coulter UK, Limited, High Wycombe, England, United Kingdom). Albumin concentrations were also measured on these auto-analysers using a colorimetric method. A sample blank absorbance reading was subtracted from the final reaction absorbance to correct any interference due to haemolysis prior to sample separation [20]. Fibrinogen and high sensitivity CRP concentrations were measured using Dade-Behring reagents on an automated Dade-Behring Nephelometer II analyser (Dade-Behring GmbH, Marburg, Germany). Previous studies had indicated small changes in measured levels of plasma analytes due to delay in plasma separation [20] ,so the analyses were adjusted for time from collection to separation, as well as for date of assay to allow for any slight analyser drift. The intra-assay coefficients of variation, based on repeat analyses of control material, were 3%–4% for all plasma analytes (apolipoprotein A_1,_ apolipoprotein B, albumin, fibrinogen, and CRP).

### Statistical analysis.

The genotypic distributions of each SNP were assessed for HWE in cases and controls separately using an exact chi-square goodness of fit test. A threshold of *p* < 0.05 was used to indicate departure from HWE. Allelic association, or LD, between SNPs was assessed using the standardised disequilibrium (D^I^) [21] and correlation *(r^2^)* [22] coefficients. The maximum likelihood estimates of haplotype frequencies and posterior probabilities of pairs of haplotypes were estimated for each individual with an expectation-maximization (E-M) algorithm under the HWE assumption using the Haplostats suite of software in R [23]. Haplotypes with an estimated frequency >1% were analyzed separately, and the remaining haplotypes were combined. A weighted kappa score was used to assess reproducibility of each of the individual SNPs studied. Analysis of variance was used to assess the associations of haplotypes with levels of cardiovascular risk factors in controls. Logistic regression adjusted for age and sex was used to estimate odds ratios for MI of each SNP and haplotype. Floated absolute risks and CIs were used to share the variance of the log odds ratios appropriately between the different genotypes or haplotypes, which allows a CI to be computed for the reference category and any group to be compared directly with any other [24,25]. The power calculations for the ISIS case-control study to detect associations of MI risk with either the rs909253 or rs10412981 SNP for the *LTA* gene were estimated using standard methods [26,27].

Finally, a meta-analysis was conducted of the associations between rs909253 or rs1041981 (which are in perfect LD in Japanese and European-descent populations) and the incidence of CHD in the ISIS study and all other published studies. For the latter studies, odds ratios for CHD were calculated from the published number of cases and controls in each of the genotype categories, either assuming an autosomal dominant model (homozygous and heterozygous vs. wild type) or recessive model (homozygous vs. heterozygous and wild type). Since the Ozaki et al. study using a recessive model was the hypothesis-generating study [5], it was not included in the combined results for the meta-analysis. The results for all remaining studies were assessed for heterogeneity and for linear trend by study size [16,28]. Summary estimates of the odds ratios from all studies were obtained by combining the separate estimates of the inverse variance–weighted log odds ratios from each study [29]. To minimise the risk of false-positive results associated with multiple comparisons, 99% confidence intervals were used for individual studies and 95% CIs for the sub-totals. All analyses were carried out using SAS version 8.2 (Cary, North Carolina, United States) and R version 1.9.1 (http://www.r-project.org).

## Abbreviations

BMI: body mass index
CHD: coronary heart disease
CI: confidence interval
CRP: C-reactive protein
HWE: Hardy-Weinberg Equilibrium
ISIS: International Study of Infarct Survival
LD: linkage disequilibrium
LTA: lymphotoxin-α
MI: myocardial infarction
SNP: single nucleotide polymorphism
